# Quantitative analysis of local microcirculation changes in early osteonecrosis of femoral head: DCE-MRI findings

**DOI:** 10.3389/fsurg.2022.1003879

**Published:** 2023-01-17

**Authors:** Pinxue Li, Congqin Xie, Yubo Liu, Zhentao Wen, Shaokui Nan, Fangyuan Yu

**Affiliations:** ^1^ School of Medicine, Nankai University, Tianjin, China; ^2^ Department of Orthopedics, The Fourth Medical Center of PLA General Hospital, Beijing, China; ^3^ National Clinical Research Center for Orthopedics, Sports Medicine & Rehabilitation, Beijing, China; ^4^ Department of Orthopedics, Handan First Hospital, Handan, China

**Keywords:** osteonecrosis of femoral head, dynamic contrast-enhanced MRI, vascular function parameters, microcirculation change, diagnostic value

## Abstract

**Aim:**

This study aims to quantitatively analyze the changes in local microcirculation in early osteonecrosis of the femoral head (ONFH) by dynamic contrast-enhanced (DCE) MRI and to explore the pathophysiological mechanisms of early ONFH.

**Patients and Methods:**

We selected 49 patients (98 hips) aged 21–59 years who were clinically diagnosed with early ONFH. A total of 77 femoral heads were diagnosed with different degrees of necrosis according to the Association Research Circulation Osseous (ARCO) staging system, and 21 femoral heads were judged to be completely healthy. All patients underwent DCE-MRI scanning. Pseudocolor images and time-signal intensity curves were generated by Tissue 4D processing software. The volume transfer constant (*K*^trans^), extracellular extravascular space, also known as vascular leakage (*V*_e_), and transfer rate constant (*K*_ep_) of healthy and different areas of necrotic femoral heads were measured on perfusion parameter maps. The differences and characteristics of these parameters in healthy and different areas of necrotic femoral heads were analyzed.

**Results:**

The signal accumulation in healthy femoral heads is lower than that of necrotic femoral heads in pseudocolor images. The time-signal intensity curve of healthy femoral heads is along the horizontal direction, while they all have upward trends for different areas of necrotic femoral heads. The mean value of *K*^trans^ of healthy femoral heads was lower than the integration of necrotic, boundary, and other areas (*F* = 3.133, *P* = .036). The *K*_ep_ value of healthy femoral heads was higher than the integration of lesion areas (*F* = 6.273, *P* = .001). The mean *V*_e_ value of healthy femoral heads was smaller than that of the lesion areas (*F* = 3.872, *P* = .016). The comparisons of parameters between different areas and comparisons among healthy areas and lesion areas showed different results.

**Conclusion:**

ONFH is a complex ischemic lesion caused by changes in local microcirculation. It mainly manifests as increased permeability of the vascular wall, blood stasis in the posterior circulation, high intraosseous pressure in the femoral head, and decreased arterial blood flow. The application of DCE-MRI scanning to quantitatively analyze the visual manifestations of microcirculation after early ONFH is an ideal method to study the microcirculation changes of necrotic femoral heads.

## Introduction

Many theories have been proposed to decipher the mechanism behind the development of osteonecrosis of the femoral head (ONFH), including the altered lipid metabolism and fat emboli theories (1, 2), intravascular coagulation theory (3), inhibition of angiogenesis theory (4), and elevated intracortical pressure theory (5). However, none of the above has been proven. To date, the pathophysiological characteristics of ONFH are still controversial. Little evidence has fully clarified the microcirculation changes in necrotic femoral heads.

With the continuous development of imaging, magnetic resonance (MR), as an examination method without ionizing radiation and with good soft tissue resolution, has received more attention from clinicians and patients. Especially in recent years, the continuous application and development of dynamic contrast-enhanced (DCE)-MRI technology and its postprocessing software have made it possible to detect the characteristics of angiogenesis and hemodynamics in tissues and directly reflect the changes in microcirculation at the lesion site (6). In DCE-MRI, the quantity of enhancement at the lesion site is closely related to the blood perfusion parameters in the local tissue, vascular enrichment, and the permeability of microvessels in the lesion tissue (6, 7).

DCE-MRI has been widely used in the evaluation of the diagnosis and treatment of tumors of various systems (8, 9). In recent years, the application of DCE-MRI to the musculoskeletal system has received more attention (10–12). Quantitative analysis of vascular function parameters obtained by DCE-MRI has been applied to femoral neck fractures (13), while there are few studies on the mechanism of ONFH (14). In this study, DCE-MRI was used to scan femoral head lesions. Vascular function parameters obtained by the postprocessing software workstation were used to quantitatively analyze the *K*^trans^, *V*_e_, and *K*_ep_ values in different areas of normal and necrotic femoral heads. Differences and characteristics of these parameters in different areas of normal and necrotic femoral heads were compared, and the changes in local microcirculation after ONFH were discussed, providing a theoretical basis for clinical treatment of the disease.

## Patients and methods

### Inclusion and exclusion criteria

In this study, 49 patients with ONFH diagnosed clinically according to Association Research Circulation Osseous (ARCO) staging (15) were analyzed. All patients underwent DCE-MRI scans of the femoral heads. There were 77 necrotic (16 in stage I and 61 in stage II) and 21 completely healthy femoral heads. The patients ranged in age from 21 to 59 years, with an average age of 39.3 years, including 40 males and 9 females. Among them, 13 patients had a history of steroid use, 25 patients had a history of long-term alcohol consumption, and 11 patients had no clear etiology.

**Figure 1:**
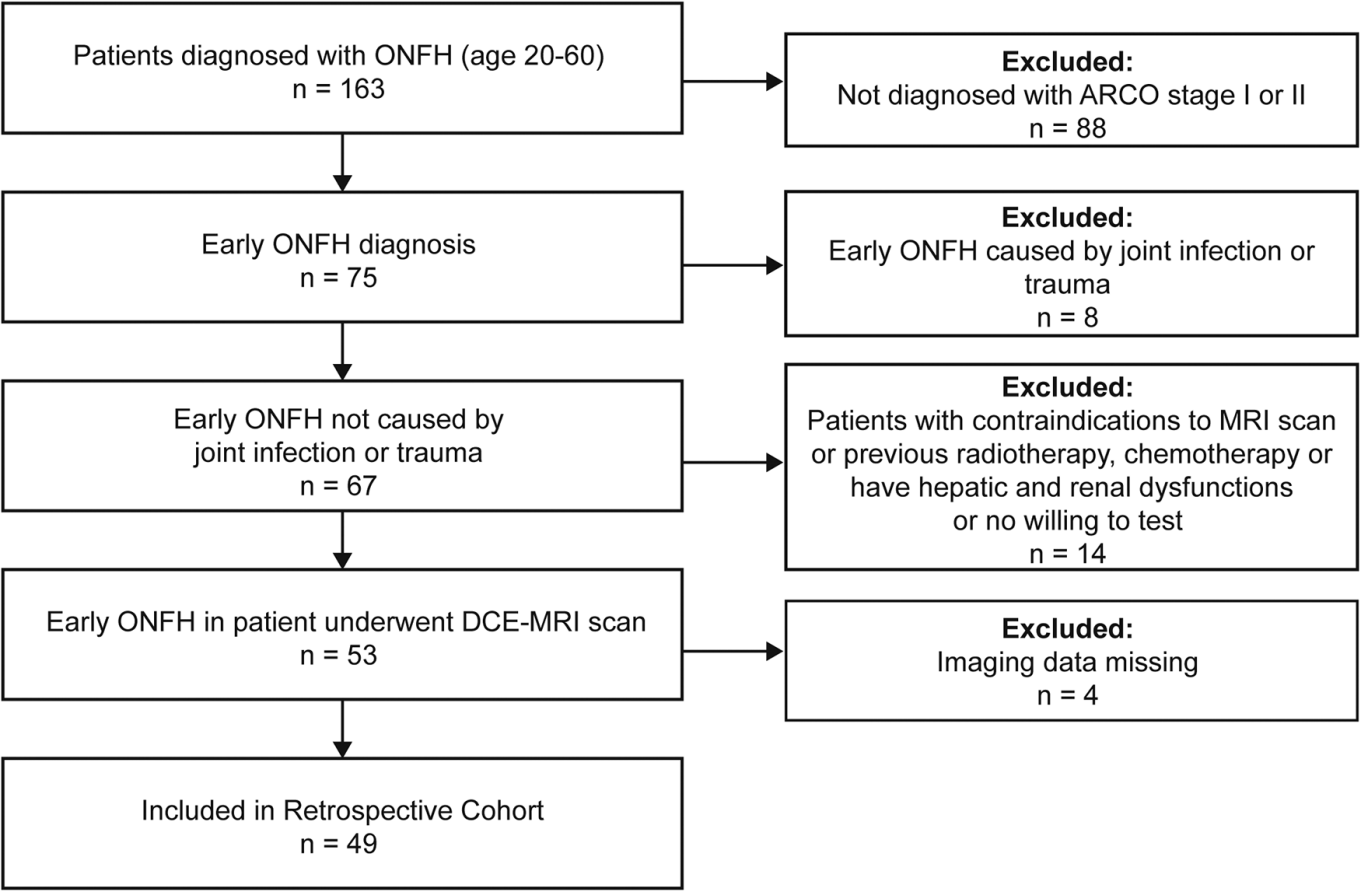
A flow diagram of included and excluded patients.

The inclusion criteria required patients with (1) a diagnosis of stage I–II femoral bone necrosis according to ARCO staging; (2) ONFH not caused by joint infection or trauma; (3) no circulatory, hepatic, renal, or other system diseases and who can tolerate DCE-MRI examination; (4) ages between 20 and 60, regardless of sex; and (5) informed consent to the relevant information and risks of this study and voluntary participation in this study. The exclusion criteria required patients with (1) contraindications to MRI examination; (2) previous radiotherapy, chemotherapy, or hepatic and renal dysfunctions; and (3) no willingness to participate in the study after fully understanding the relevant situation and risks of the study. A flow diagram of included and excluded patients is provided in Figure 1.

### DCE-MRI scanning

Dynamic enhanced MRI T1WI, T2WI, and T1 scans were performed on all patients. A Siemens Magnetom Skyra 3.0T superconducting MRI scanner (Siemens Healthineers GmbH, Erlangen, Germany) with Body 18 A 3T Tim Coil (Siemens Healthineers GmbH, Erlangen, Germany) was used. Patients were placed in a supine position with their heads first. Body 18 A 3T Tim Coil was used to cover the abdomen. The scanning center was determined to be 3 cm above the symphysis pubis. The coil was firmly fixed with the bandage to avoid artifacts.

Start with a routine hip scan: Axial T1WI [time of repetition (TR) 868 ms, time of echo (TE) 10 ms, number of slices 19, slice thickness 4.0 mm, field of view (FOV) 300 mm, number of excitation (NEX) 1, flip angle (FA) 145°], T2WI-FS (TR 3600 s, TE 77 ms, slice thickness 4.0 mm, NEX 2) sequence, Coronal T2WI-FS (TR 2300 ms,TE 33 ms, slice thickness 3.0 mm) sequence; Axial T1 dynamic enhanced scanning: T1 mapping (TR/TE 4.09 ms/1.39 ms; FOV 300 mm; slice thickness 3.5 mm; slice gap 0.3 mm, NEX 1; FA 2°/15°). Then, T1 continuous enhanced sequences (TR/TE 4.83 ms/1.87 ms, FOV 300 mm, slice thickness 3.5 mm, slice gap 0.3 mm, NEX 1, FA 15°, total scanning phases 75, total scanning time 26 min) were used in the axial position. At the end of the 5th phase of data collection, the contrast medium gadopentetate dimeglumine (Gd-DTPA) (flow rate: 2.5 mL/s, 0.2 mmol/kg) was injected into the upper elbow vein at high pressure, followed by 20 mL of normal saline, with a flow rate of 5.0 mL/s. Scan without intermittency.

### Image processing

The original images obtained by DCE-MRI were sent to the Siemens Syngo (Siemens Healthineers GmbH, Erlangen, Germany) workstation. The images of the first 75 stages were selected, after contrasting medium injection, and the data were postprocessed by Tissue 4D software (Siemens Healthineers GmbH, Erlangen, Germany).

Image processing of normal femoral heads: The axial plane is the main measuring plane. The slice that can show the largest diameter of the femoral head is regarded as the best slice. The whole femoral head was selected as the region of interest (ROI), excluding the cortex. After computer processing, the time-signal intensity curve was generated to display the pseudocolor image of the ROI. The vascular function parameters *K*^trans^, *V*_e_, and *K*_ep_ values were calculated.

Image processing of necrotic femoral heads: (1) The axial plane is the main measuring plane. The slice with the largest lesion area is considered the best slice after image motion correction. The whole femoral head was selected as the ROI, excluding the cortex. ROI-1 (healthy femoral heads) was defined as the normal femoral head area. ROI-2 (necrotic area) was defined as the focal signal change in the subchondral area of the anterolateral weight-bearing area of the femoral head with segmental hyposignal on T1WI or “double line sign” on T2WI. ROI-3 (the boundary area or repair area) was defined as the boundary area of the necrotic area. ROI-4 (edema area) was defined as the focal signal change in the femoral head with a high signal on T2WI. After computer processing, the time-signal intensity curve was generated to display the pseudocolor image of the ROI. The vascular function parameters *K*^trans^, *V*_e_, and *K*_ep_ values were calculated. (2) The axial plane is the main measuring plane. The best slice is determined by image motion correction and is the slice that best shows lesions. The necrotic area, boundary area, and other areas of the necrotic femoral heads were selected as target sites. ROIs can be round or oval, including as many target areas as possible. After computer processing, the time-signal intensity curve was generated to display the pseudocolor image of the ROI. The vascular function parameters *K*^trans^, *V*_e_, *K*_ep_, and iAUC were calculated.

All postprocessing of perfusion MRI images was completed by two imaging physicians with senior professional titles.

### Statistical process

All tests were performed using SPSS 18.0 (IBM Corp, Armonk, NY, USA). All measurement data were tested for normality and homogeneity of variance by a single-sample *K*–*S* test and the Levene variance homogeneity test. Independent sample *T* tests were used to compare the overall parameters of the normal and necrotic femoral heads. Analysis of variance (ANOVA) was used to compare the parameters of normal femoral heads with those of necrotic, boundary, and other areas of necrotic femoral heads. The least-significant difference (LSD) method was used for data with homogeneity of variance, and Tamhane's T2 (M) method was used for data with unequal variance. For data with unequal variances, the independent sample *T* test was used to further demonstrate the comparison among groups.

All measurement data are expressed as the mean ± standard deviation. The significance level was *α* = 0.05, and a *P*-value < .05 was considered significant in all analyses.

## Results

All 49 patients who underwent DCE-MRI scanning received high-quality images that could be used for postprocessing. No errors were found in the processing of the Tissue 4D software, and there was no obvious artifact affecting the measurement of parameters and no obvious error in the obtained quantitative parameters.

### Generation of pseudocolor images and time-signal curves and comparisons between healthy and necrotic femoral heads

**Figure 2:**
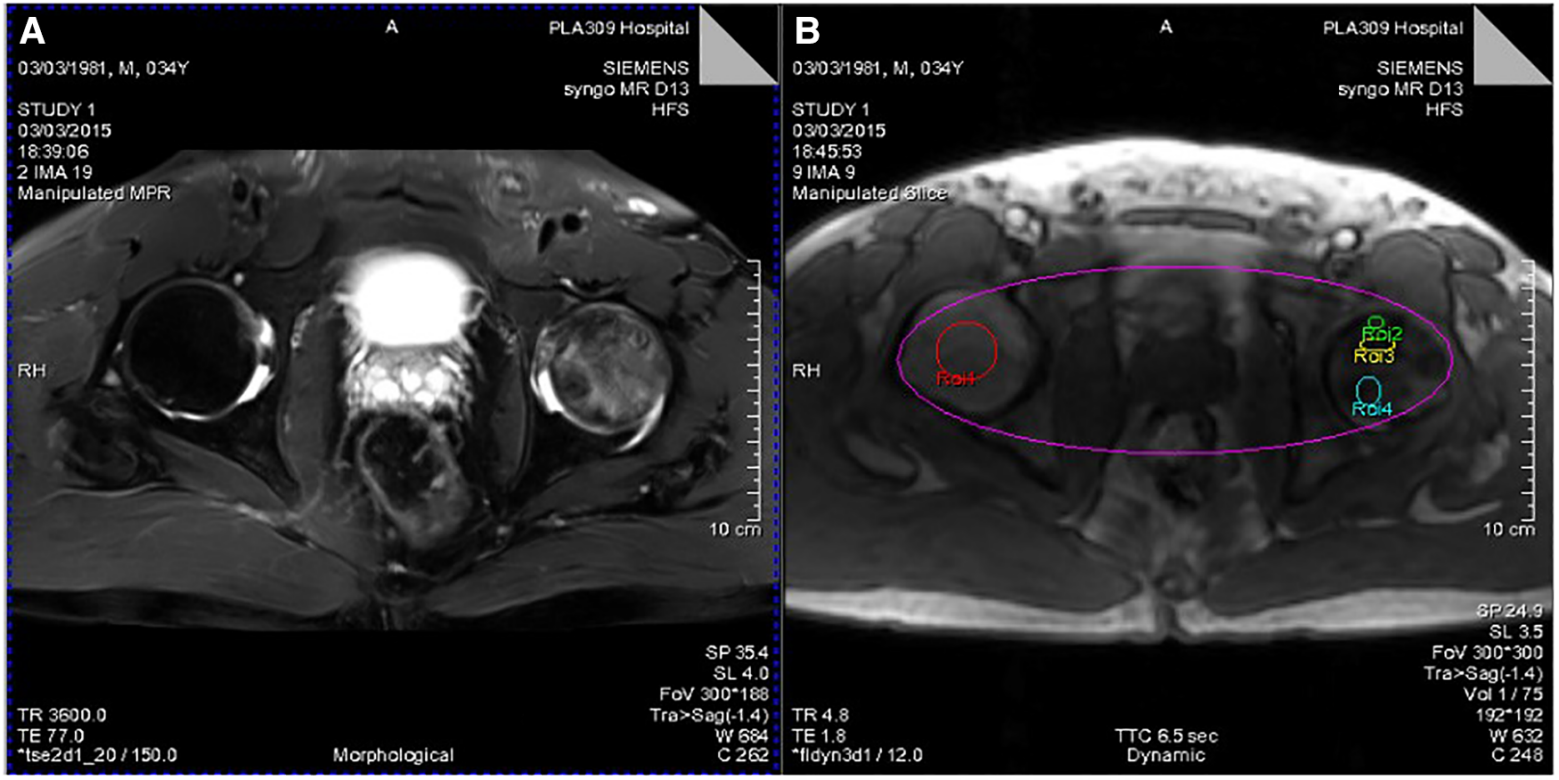
(**A**) T2WI lipid suppression image showing necrosis and edema of the left femoral head. (**B**) Different ROIs selected by Tissue 4D processing software (ROI-1, healthy femoral heads; ROI-2, necrotic area; ROI-3, boundary area; ROI-4, edema area).

**Figure 3:**
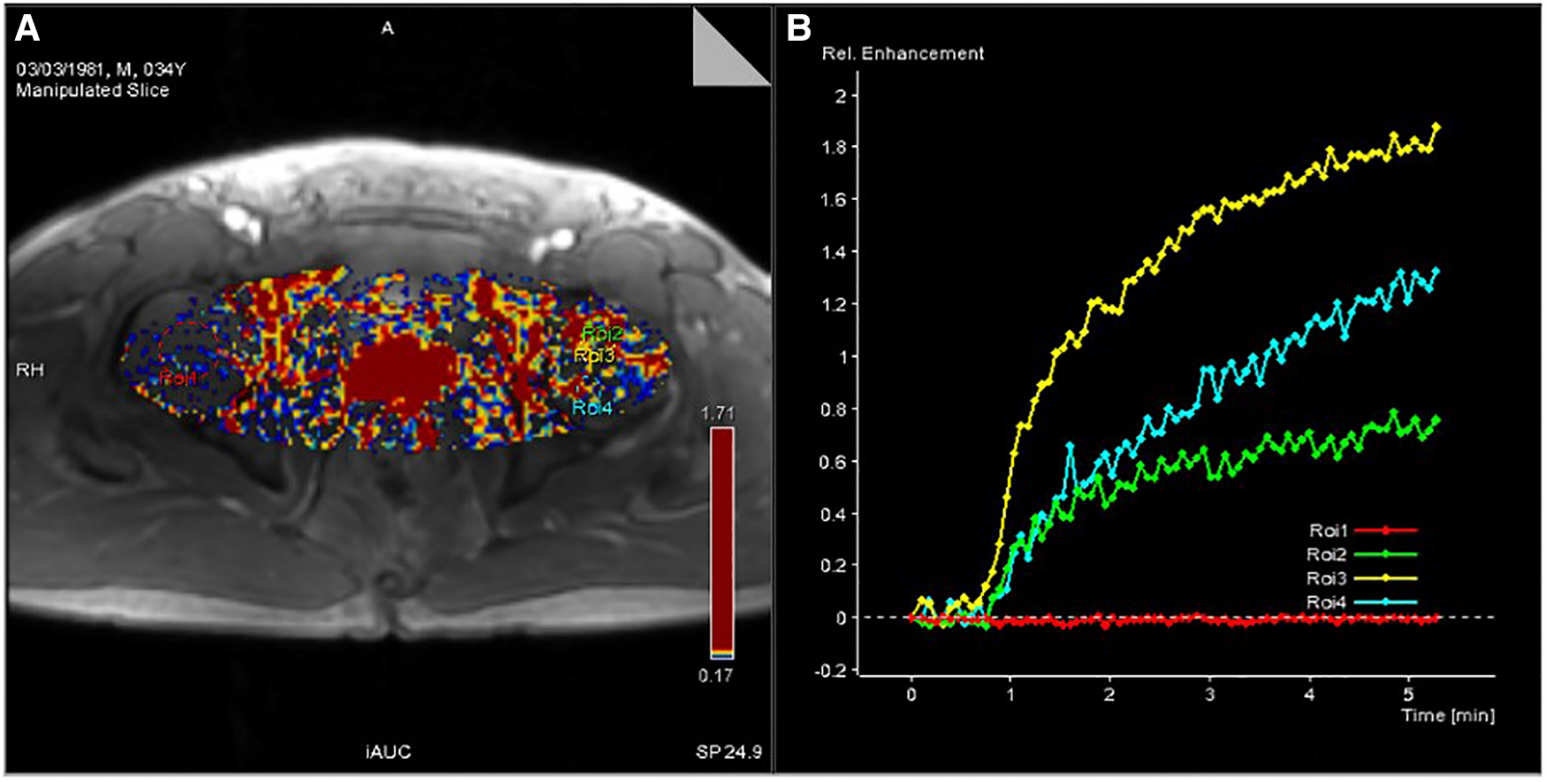
(**A**) Pseudocolor image processed by Tissue 4D software. (**B**) Time-signal intensity curve of ROIs of the four selected regions of the femoral head generated by Tissue 4D software.

Pseudocolor images and time-signal intensity curves of normal and necrotic femoral heads can be obtained by using DCE-MRI scanning technology and Tissue 4D processing software (Figures 2, 3).

The mean value of *K*^trans^ healthy femoral heads was lower than the integration of necrotic, boundary, and other areas (*F* = 3.133, *P* = .036). The *K*_ep_ value of healthy femoral heads was higher than the integration of lesion areas (*F* = 6.273, *P* = .001). The mean value of *V*_e_ in healthy femoral heads was smaller than that of the lesion areas (*F* = 3.872, *P* = .016).

### Comparison of quantitative parameters among the normal area, necrotic area, edema area, and boundary area of the femoral heads

**Figure 4:**
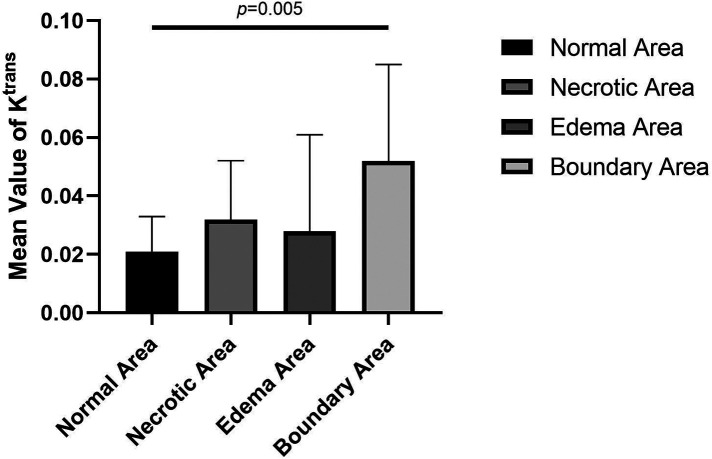
Comparison of mean values of *K*^trans^ of normal, necrotic, edema, and boundary areas of femoral heads in patients with ONFH.

**Figure 5:**
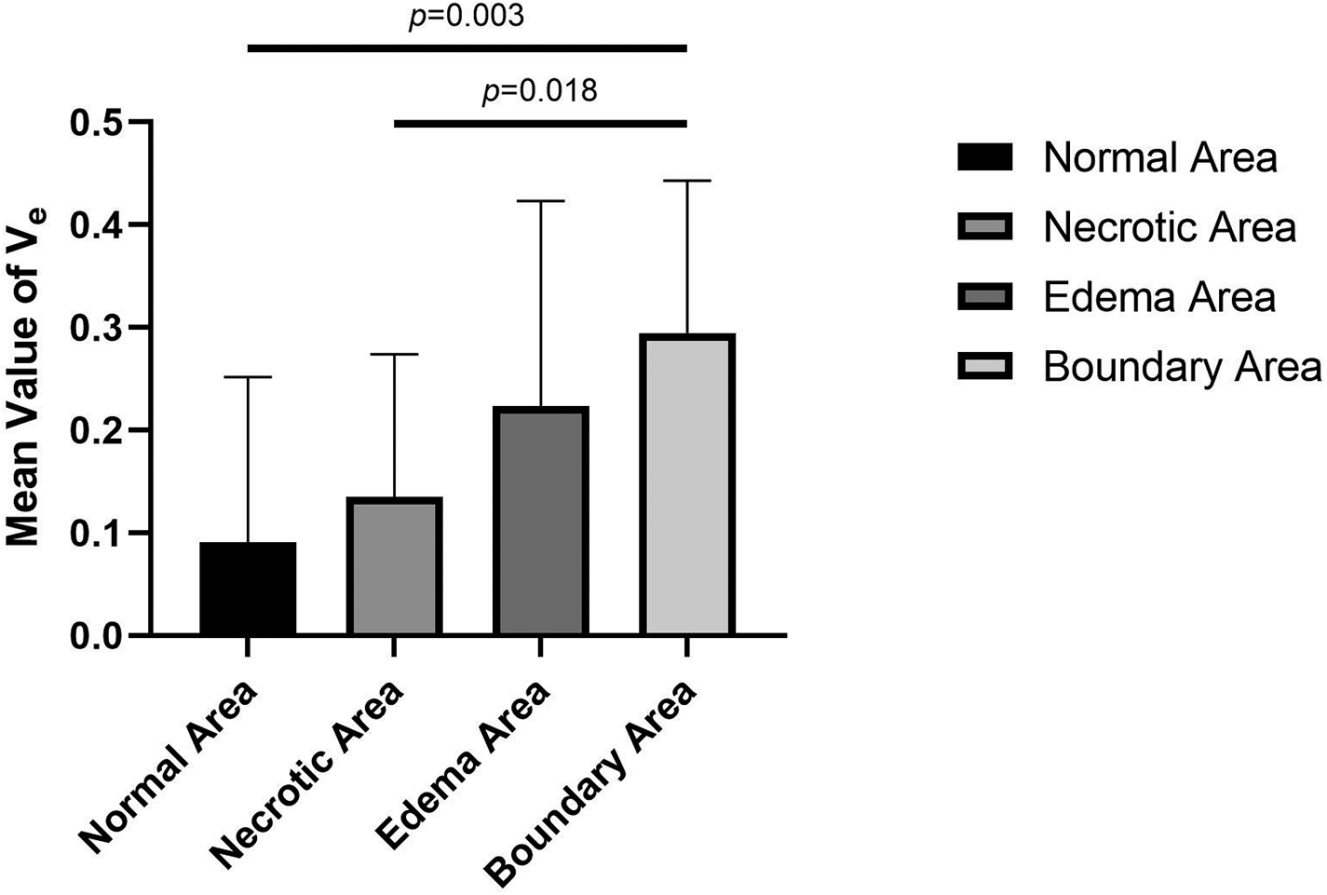
Comparison of the mean *K*_ep_ values in normal, necrotic, edema and boundary areas of the femoral heads in patients with ONFH.

**Figure 6:**
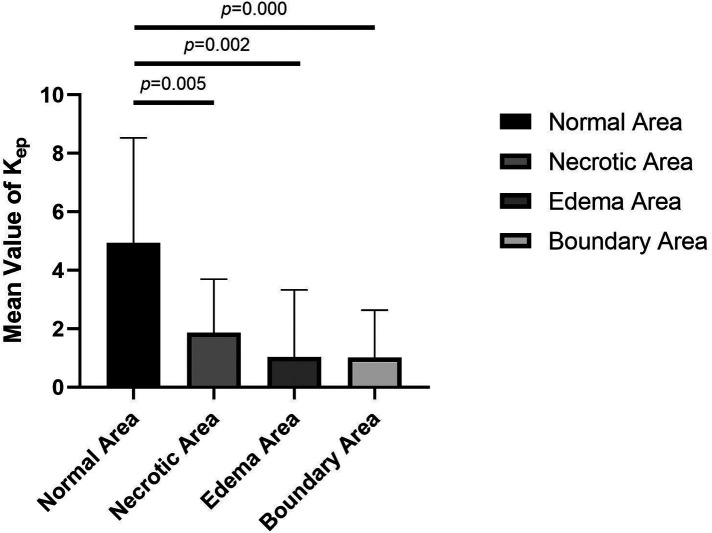
Comparison of the mean values of *V*_e_ in normal, necrotic, edema, and boundary areas of femoral heads in patients with ONFH.

The values of *K*^trans^, *K*_ep_, and *V*_e_ in the normal area, necrosis area, edema area, and boundary area of the femoral heads conformed to a normal distribution and homogeneity of variance. One-way ANOVA revealed statistically significant effects in *K*^trans^ (*F* = 3.133, *P* = .036), *K*_ep_ (*F* = 6.273, *P* = .001), and *V*_e_ (*F* = 3.872, *P* = .016) among the normal area, necrotic area, edematous area, and boundary area of the femoral heads. The mean values of *K*^trans^ and *V*_e_ in the boundary area were higher than those in the necrotic area, edema area, and normal area of the femoral heads. The mean values of *K*^trans^ and *V*_e_ in the normal area were lower than those in the necrotic area and edema area. In the necrotic area, *K*^trans^ was higher and *V*_e_ was lower than those in the edema area. The *K*_ep_ value was the highest in the normal area, followed by the necrotic area, the edema area, and the lowest in the boundary area. In pairwise comparisons, the values of *K*^trans^, *K*_ep_, and *V*_e_ were statistically significant between the normal and boundary areas. The *K*_ep_ values were statistically significant in comparisons between normal and necrotic areas and normal and edema areas. The values of *V*_e_ were statistically significant between the necrotic and boundary areas. There was no statistical significance in pairwise comparisons among other groups (*P* > .05) (Tables 1, 2 and Figures 4–6).

**Table 1:** Results of mean values of vascular function parameters in the normal area, necrotic area, edema area, and boundary area of femoral heads in patients with ONFH.

T1 parameters^a^	Normal area	95% CI	Necrotic area	95% CI	Edema area	95% CI	Boundary area	95% CI
	A, *n* = 43		B, *n* = 64		C, *n* = 49		D, *n* = 53	
*K* ^trans^	0.021 ± 0.012	0.013–0.028	0.032 ± 0.020	0.018–0.443	0.028 ± 0.033	0.003–0.059	0.052 ± 0.033	0.031–0.073
*K*_ep_	4.926 ± 3.606	2.634–7.218	1.872 ± 1.820	0.715–3.028	1.027 ± 2.303	0.000–3.158	1.016 ± 1.620	0.000–0.389
*V*_e_	0.091 ± 0.161	0.000–0.193	0.135 ± 0.139	0.045–0.224	0.224 ± 0.199	0.040–0.408	0.295 ± 0.148	0.201–0.389

ONFH, osteonecrosis of the femoral head.

^a^ The unit of *K*^trans^ and *K*_ep_ is min^−1^; *V*_e_ is constant without unit.

**Table 2:** Results of pairwise comparisons among the normal area, necrotic area, edema area, and boundary area of femoral heads in patients with ONFH.

T1 parameters	A and B	A and C	A and D	B and C	B and D	C and D
	*P*-value	*P*-value	*P*-value	*P*-value	*P*-value	*P*-value
*K* ^trans^	*P* = .334	*P* = .549	*P* = .005^a^	*P* = .815	*P* = .061	*P* = .061
*K*_ep_	*P* = .005^a^	*P* = .002^a^	*P* = .000^a^	*P* = .479	*P* = .404	*P* = .993
*V*_e_	*P* = .500	*P* = .086	*P* = .003^a^	*P* = .245	*P* = .018^a^	*P* = .350

A, normal area; B, necrotic area; C, edema area; D, boundary area; ONFH, osteonecrosis of the femoral head.

^a^ The difference between the two groups were statistically significant.

### Comparison of quantitative parameters between healthy and different areas of necrotic femoral heads

The values of *K*^trans^, *K*_ep_, and *V*_e_ in healthy femoral heads conformed to a normal distribution. Compared with necrotic and boundary areas, healthy femoral heads had significant differences in the mean values of *K*^trans^, *K*_ep_, and *V*_e_. Compared with the normal area of necrotic femoral heads, only the *K*^trans^ difference was statistically significant, and when compared with the edema area, the *K*_ep_ and *V*_e_ value differences were statistically significant (Tables 3, 4).

**Table 3:** Mean values of *K*^trans^, *K*_ep_, and *V*_e_ in healthy femoral heads and different areas of necrotic ones.

T1 parameters	Healthy femoral heads	Normal area	Necrotic area	Edema area	Boundary area
*K* ^trans^	0.012 ± 0.002	0.021 ± 0.012	0.032 ± 0.020	0.028 ± 0.033	0.052 ± 0.033
*K*_ep_	4.164 ± 1.798	4.926 ± 3.606	1.872 ± 1.820	1.027 ± 2.303	1.016 ± 1.620
*V*_e_	0.021 ± 0.014	0.091 ± 0.161	0.135 ± 0.139	0.224 ± 0.199	0.295 ± 0.148

**Table 4:** Results of pairwise comparisons between healthy femoral heads and different areas of necrotic ones, respectively (independent samples *T* test).

T1 parameters	A and E		B and E		C and E		D and E	
	*t*-value	*P*-value	*t*-value	*P*-value	*t*-value	*P*-value	*t*-value	*P*-value
*K* ^trans^	*t* = 2.265	*P* = .041^a^	*t* = 1.979	*P* = .010^a^	*t* = 1.029	*P* = .328	*t* = 2.579	*P* = .021^a^
*K*_ep_	*t* = 0.444	*P* = .664	*t* = −2.374	*P* = .031^a^	*t* = −2.532	*P* = .030^a^	*t* = −3.539	*P* = .003^a^
*V*_e_	*t* = 0.940	*P* = .362	*t* = 2.776	*P* = .017^a^	*t* = 2.680	*P* = .036^a^	*t* = 4.045	*P* = .001^a^

A, normal area; B, necrotic area; C, edema area; D, boundary area; E, healthy femoral heads.

^a^ The difference between the two groups were statistically significant.

## Discussion

DCE-MRI is a widely used examination method to detect changes in microcirculation and blood circulation in relevant parts of the musculoskeletal system. The time-signal intensity curve and pseudocolor image of the target site can be obtained after processing by Tissue 4D software to semi-quantitatively describe the microcirculation characteristics of the target site. The quantitative analysis of DCE-MRI is based on the two-compartment Tofts–Kermode model, and then a series of corresponding mathematical calculations were performed to obtain the vascular functional parameters that could quantitatively reflect the changes in microcirculation and blood circulation at the target site (16–19). The parameters include the volume transfer constant (*K*^trans^), extracellular extravascular space, also known as vascular leakage (*V*_e_) and transfer rate constant (*K*_ep_); *K*^trans^ represents the transport volume of small molecule contrast medium diffused from intravascular to extravascular space per unit time, which is mainly affected by microcirculation structure, blood flow, the transport process of contrast medium through the blood vessel wall, and the diffusion process of contrast medium in intercellular space. *K*_ep_ represents the amount of contrast medium returned to the blood vessel after tissue diffusion within a unit time.

### Analysis of time-signal intensity curves and pseudocolor images obtained by DCE-MRI scanning

The pseudocolor image processed by Tissue 4D software (Figure 3A) indicates that the necrotic femoral head presents a high accumulation of enhanced signals, while the healthy femoral head presents a low accumulation of enhanced signals. From the many hypotheses about the mechanism of ONFH, the pathophysiological development will cause changes in the microcirculation of the femoral head, such as blood stasis in the posterior circulation, venous obstruction, reduced arterial blood supply, and intraosseous high pressure in the femoral head. In the pseudocolor image (Figure 3A), the high accumulation of enhancement signals after ONFH also confirmed the changes in microcirculation in the hypotheses above. Due to blood stasis in the posterior circulation after osteonecrosis, blocked venous return and high pressure in the femoral head, the permeability of the vascular wall of the microcirculation was changed. The large amount of contrast medium exuded from the blood vessels could not be effectively excluded, resulting in a high accumulation of enhanced signals. However, in healthy femoral heads, microcirculation, the vascular wall, and intraosseous pressure were all under physiological conditions, and contrast medium showed normal intake and excretion, resulting in a low accumulation of enhanced signals.

Figure 3B shows the time-signal intensity curve generated by Tissue 4D software processing the selected ROIs. The red curve is the time-signal intensity curve of ROI-1 selected in the healthy femoral head, which fluctuates in the horizontal direction, indicating the normal uptake and elimination of contrast medium. The green curve is the time-signal intensity curve of ROI-2 in the necrotic area of the selected left femoral head, which shows a slow upward trend, indicating a decrease in microcirculation vessels, poor blood circulation, less contrast medium intake, slow elimination, yet still slow accumulation of contrast medium. The yellow curve is the time-signal intensity curve of ROI-3 in the boundary area (repair area), which is located at the edge of the necrotic area of the femoral head and has the fastest upward trend and the highest signal accumulation. Due to capillary angiogenesis and high vascular permeability due to incomplete development of the neonatal capillary wall, the boundary area has the best blood supply, the fastest arrival, and the most content of contrast medium compared with other areas. Nevertheless, due to blood stasis in the microcirculation, the contrast medium is not eliminated quickly enough to generate the fastest-rising time-signal intensity curve. The blue curve is the time-signal intensity curve of ROI-4 in the edema area of the femoral head selected, which has a faster upward trend than in the necrotic area, indicating high vascular permeability and better blood supply than that in the necrotic area. Due to intercellular edema, and blood stasis in the microcirculation, the accumulation of contrast medium is relatively difficult to eliminate.

### Analysis of changes in microcirculation and blood supplies among different areas of necrotic femoral heads

In this study, DCE-MRI quantitative analysis showed that the mean value of *K*^trans^ was the lowest in the normal area, followed by the edema area and necrotic area, and the highest value appeared in the boundary area (repair area) of necrotic femoral heads (Table 1 and Figure 4).

*K*^trans^ denotes the transport volume of small molecule contrast medium diffused from intravascular to extravascular within a unit time, which is influenced primarily by microcirculation structure, blood flow, contrast medium diffusion through vascular walls and intercellular spaces. Previous studies have found that an abnormal increase in the *K*^trans^ value is believed to be related to the microvascular density and vascular permeability of the detected site (20). Glueck et al. (21) proposed in their study that mutations in the eNOS gene are positively correlated with ONFH and could lead to incomplete structures in the vascular wall during neovascularization. The mean *K*^trans^ value of the boundary areas of necrotic femoral heads was higher than that of the normal areas, and statistical significance was found in the pairwise comparison. This result proves that there are changes in the structures and permeability of vessel walls, including neovasculature, in necrotic femoral heads, which is in accordance with the results of Glueck et al.

The value of *K*_ep_ represents the amount of contrast medium flowing back through the vessels per unit time after diffusion into tissues, and it is not only related to the vascular permeability of microcirculation but also to the status of posterior circulation. When the status of the posterior circulation changes, it will directly affect the osmotic pressure on both sides of the vascular wall and then affect the reflux rate of the contrast medium and the *K*_ep_ value. Abnormally elevated *K*_ep_ values are associated with microvascular density and vascular permeability (20), and the *K*_ep_ value often increases or decreases simultaneously with *K*^trans^ in most instances. In this study, the mean value of *K*^trans^ in the boundary areas was higher than that in the normal areas, but the mean value of *K*_ep_ was lower in the boundary areas and in the necrotic and edema areas (Table 1 and Figure 5). In pairwise comparisons, differences in the mean values of *K*_ep_ in the normal area and in other lesion areas were statistically significant, which is not exactly the same as the distribution variations in tumor tissues and normal tissues (14, 22). When ONFH develops, the permeability of microcirculation vessels will increase, but the *K*_ep_ value will become lower than that in healthy tissues, which is consistent with the theories and hypotheses of ONFH that, in the later stage of the disease, changes in microcirculation manifest blood stasis in the posterior circulation, blocked venous return, and result in increased intraosseous pressure. The higher osmotic pressure in blood vessels in lesion areas prevents the effective reflux of contrast medium so that the *K*_ep_ values become lower than those in the normal area.

*V*_e_ stands for extracellular extravascular space and has a relationship with *K*^trans^ and *K*_ep_ values as follows: *V*_e _= *K*^trans^/*K*_ep_. Therefore, the change in the *V*_e_ value is closely related to the *K*^trans^ and *K*_ep_ values. In this study, the variation trend of the mean value of *V*_e_ gradually increased from the normal area, necrotic area, and edema area to the boundary area, which was consistent with the variation trend of *K*^trans^ and *K*_ep_ values. In pairwise comparisons of the mean values of *V*_e_ in different areas, there were statistically significant differences between normal and boundary areas and between necrotic and boundary areas. No significant differences were identified among other pairings. The change in the *V*_e_ value indirectly confirms that the above description of the pathophysiological changes in the mechanism of ONFH is correct.

### Comparative analysis of changes in microcirculation and blood supplies between healthy femoral heads and different areas of necrotic femoral heads

In this study, the differences in vascular function parameters between healthy femoral heads and different areas of necrotic femoral heads were further compared. It indicates that there was no statistically significant difference in mean values of *K*^trans^ between healthy femoral heads and edema areas of necrotic femoral heads, whereas they both were lower than those in other areas. All pairwise comparisons of *K*^trans^ between healthy femoral heads and each area of necrotic femoral heads showed statistically significant differences (Tables 3, 4). There was no statistical significance between healthy femoral heads and edema areas, which may be attributed to the relatively small sample size of edema areas. In the progression of ONFH, the microcirculation and vascular wall will change compared with healthy femoral heads, mainly manifested as increased permeability of the vascular wall.

The mean value of *K*_ep_ in healthy femoral heads is greater than those in necrotic, edema, and boundary areas of necrotic femoral heads, while *V*_e_ is smaller than the three areas. When healthy femoral heads and the three areas were compared in pairs, the results showed that the differences in *K*_ep_ and *V*_e_ values were all statistically significant. In the comparison between healthy femoral heads and normal areas in necrotic femoral heads, differences in the values of *K*_ep_ and *V*_e_ were not statistically significant. The results again confirmed that in the process of ONFH, the microcirculation changes are mainly manifested in increased permeability of the vascular wall, blood stasis in the posterior circulation, high intraosseous pressure in the femoral head and decreased arterial blood flow.

### Limitation

In this study, quantitative data between different ARCO stages were not compared and analyzed. On the other hand, the judgment of different femoral head necrosis areas, such as necrotic areas and edema areas, depends more on visual and empirical judgment and lacks objective and unified standards. Moreover, different causes, such as alcohol-induced and steroid-induced osteonecrosis of the femoral head, were not analyzed and compared by microcirculation of the femoral head in this study. Therefore, quantitative analysis of the mechanism of femoral head necrosis using vascular function parameters obtained from DCE-MRI needs to be further explored.

## Conclusion

We believe that this is the first and largest study applying DCE-MRI to early ONFH in human patients. Vascular function parameters were compared between healthy femoral heads and areas in necrotic femoral heads, which quantitively described and directly proved the pathophysiological mechanism of early ONFH. The main manifestations include increased permeability of the vascular wall, blood stasis in the posterior circulation, high intraosseous pressure in the femoral head, and decreases in arterial blood flow. All the results provide a theoretical basis for the clinical treatment of early ONFH.

## Data Availability

The original contributions presented in the study are included in the article/Supplementary Materials, further inquiries can be directed to the corresponding author/s.
